# rhBMP-2 induces terminal differentiation of human bone marrow mesenchymal stromal cells only by synergizing with other signals

**DOI:** 10.1186/s13287-024-03735-y

**Published:** 2024-04-29

**Authors:** Neda Kathami, Carolina Moreno-Vicente, Pablo Martín, Jhonatan A. Vergara-Arce, Raquel Ruiz-Hernández, Daniela Gerovska, Ana M. Aransay, Marcos J. Araúzo-Bravo, Sandra Camarero-Espinosa, Ander Abarrategi

**Affiliations:** 1 Center for Cooperative Research in Biomaterials (CIC biomaGUNE), Basque Research and Technology Alliance (BRTA), 20014 Donostia-San Sebastian, Spain; 2 POLYMAT, University of the Basque Country (UPV-EHU), 20018 Donostia-San Sebastian, Spain; 3 Complutense University of Madrid, 28040 Madrid, Spain; 4 Computational Biology and Systems Biomedicine, Biodonostia Health Research Institute, 20014 San Sebastián, Spain; 5 Center for Cooperative Research in Biosciences (CIC bioGUNE), Basque Research and Technology Alliance (BRTA), 48160 Derio, Spain; 6 Centro de Investigación Biomedica en Red de Enfermedades Hepáticas y Digestivas (CIBERehd), Instituto de Salud Carlos III, Madrid, Spain; 7 Department of Cell Biology and Histology, Faculty of Medicine and Nursing, University of Basque Country (UPV/EHU), 48940 Leioa, Spain; 8 IKERBASQUE, Basque Foundation for Science, 48009 Bilbao, Spain; 9 Regenerative Medicine and Disease Models Laboratory, Center for Cooperative Research in Biomaterials (CIC biomaGUNE), Paseo Miramón, 194, 20014 Donostia, Gipuzkoa Spain

**Keywords:** rhBMP-2, Mesenchymal stem cells, Differentiation, Osteogenesis, Chondrogenesis, Adipogenesis

## Abstract

**Background:**

Recombinant human bone morphogenetic protein 2 (rhBMP-2) and human bone marrow mesenchymal stromal cells (hBM-MSCs) have been thoroughly studied for research and translational bone regeneration purposes. rhBMP-2 induces bone formation in vivo, and hBM-MSCs are its target, bone-forming cells. In this article, we studied how rhBMP-2 drives the multilineage differentiation of hBM-MSCs both in vivo and in vitro.

**Methods:**

rhBMP-2 and hBM-MSCs were tested in an in vivo subcutaneous implantation model to assess their ability to form mature bone and undergo multilineage differentiation. Then, the hBM-MSCs were treated in vitro with rhBMP-2 for short-term or long-term cell-culture periods, alone or in combination with osteogenic, adipogenic or chondrogenic media, aiming to determine the role of rhBMP-2 in these differentiation processes.

**Results:**

The data indicate that hBM-MSCs respond to rhBMP-2 in the short term but fail to differentiate in long-term culture conditions; these cells overexpress the rhBMP-2 target genes *DKK1*, *HEY-1* and *SOST* osteogenesis inhibitors. However, in combination with other differentiation signals, rhBMP-2 acts as a potentiator of multilineage differentiation, not only of osteogenesis but also of adipogenesis and chondrogenesis, both in vitro and in vivo.

**Conclusions:**

Altogether, our data indicate that rhBMP-2 alone is unable to induce in vitro osteogenic terminal differentiation of hBM-MSCs, but synergizes with other signals to potentiate multiple differentiation phenotypes. Therefore, rhBMP-2 triggers on hBM-MSCs different specific phenotype differentiation depending on the signalling environment.

**Supplementary Information:**

The online version contains supplementary material available at 10.1186/s13287-024-03735-y.

## Background

Bone is a complex structure composed of multiple specialized cell types, such as mesenchymal, endothelial, nervous, and hematopoietic cells. After an injury, bone has a well-known intrinsic regenerative capacity and is able to reestablish homeostasis among these cell types in the injured area. Mesenchymal cells are considered to be the effectors of bone regeneration, and during this process, they are influenced by multiple sequential and overlapping signals, which together trigger proper bone healing. However, the tissue-intrinsic regenerative potential often is not enough to drive and heal bone defects and/or injuries. In this context, bone tissue engineering is a translational field that aims to apply basic knowledge of bone signals and cells to potentiate bone formation processes [1].

The family of bone morphogenetic proteins (BMPs) was identified in bone extracts. They participate in multiple molecularly complex biological processes, such as embryogenesis, organogenesis and tissue repair. In the bone tissue, BMPs are known as key factors for skeletal development and regeneration processes [2]. Due to its ability to form bone tissue in vivo, recombinant human BMP-2 (rhBMP-2) has been thoroughly studied for research and translational therapeutic purposes [2, 3]. The biological activity of rhBMP-2 has been assessed in multiple contexts. In vitro, in short-term cell culture studies, active rhBMP-2 triggers global changes in the gene expression profile of some mesenchymal cells, including the upregulation of early osteogenic differentiation markers, such as alkaline phosphatase (ALP) [4]. On the other hand, in vivo, the ectopic or orthotopic implantation of biologically active rhBMP-2 in combination with proper rhBMP2-carrier materials locally triggers mature bone formation, which requires mesenchymal cell multilineage differentiation [5].

Specifically, bone marrow mesenchymal stroma cells (BM-MSCs), along with periosteal cells, have been postulated as the cells driving the bone formation during regeneration [6]. At the translational level, human BM-MSCs (hBM-MSCs) have been thoroughly used as a valid cell model in bone regeneration and implantable approaches [7], as well as in combination with rhBMP-2 [8]. Multiple cell parameters of hBM-MSCs exhibit interdonor and intradonor variability; therefore, the differentiation ability of each cell culture must be assessed before the intended therapeutic usage [9]. In vitro, different methods and media formulations have been developed to induce and test the terminal in vitro osteogenic, adipogenic and chondrogenic differentiation ability of hBM-MSCs. Mostly, each differentiation phenotype is achieved by the supplementation of the culture media with multiple specific chemicals and/or growth factors, each triggering different molecular pathways to synergically achieve the expected cell phenotype [10–12].

Most of the reports studying rhBMP-2 during bone regeneration are focused only on its osteogenic ability. The few studies addressing the role of rhBMP-2 in inducing chondrogenic and adipogenic differentiation of hBM-MSCs pointed a key relevance of integrating rhBMP-2 with other signals to successfully achieve terminal differentiation [13–15].

In this work, we studied whether rhBMP-2 drives in vitro multilineage differentiation in cultured hBM-MSCs and how and under what conditions. First, it was assessed that rhBMP-2 triggered in vivo bone formation and multilineage differentiation in the tested hBM-MSCs. Then, short-term in vitro treatment of hBM-MSCs with rhBMP-2 was used to induce the expression of specific differentiation markers. After rhBMP-2 treatment for 28 days, a negative feedback loop was activated, which blocked the rhBMP-2-related global rearrangement of gene expression. Finally, multiple differentiation media were supplemented with rhBMP-2, and rhBMP-2 was shown to synergize with the other agents to potentiate terminal osteogenic, adipogenic and chondrogenic phenotypes in vitro. Taken together, these findings suggest that rhBMP-2 alone is unable to induce in vitro hBM-MSC terminal differentiation but synergizes with other differentiation signals to facilitate the terminal differentiation of multiple phenotypes in vitro, resembling the mesenchymal phenotypes observed in in vivo bone formation processes.

## Materials and methods

### Cell cultures

Healthy human donor bone marrow mesenchymal stromal cells (hBM-MSCs) were obtained from different commercial providers (Lonza, Cat.# PT-2501; Cambrex, Cat.# PT-2501; Millipore, Cat.# SCR108; Inbiobank, Cat.# hMSC). All donor cells were obtained under informed consent and were tested for the absence of endothelial and hematopoietic markers; presence of specific mesenchymal markers; and ability to differentiate into osteogenic, chondrogenic and adipogenic phenotypes, as defined by the International Society for Cellular Therapy (ISCT) criteria [16].

The cells were expanded according to the manufacturer’s instructions in MSCBM mesenchymal cell proliferation media (Lonza, Cat.# PT-3001; or Stem Cells Tech, Cat.# 5411), which included basal medium, mesenchymal stem cell stimulatory supplements, L-glutamine and antibiotics (Pen/strep). Subcultures were performed when the cells reached 80% confluence by conventional enzymatic cell detachment with 0.25% trypsin-EDTA (Gibco, Cat.# 25200056), followed by cell counting and seeding at 6000 cells/cm^2^ for subculture or 100,000 cells/well in 6-well plates (Corning, Cat.# 3516) for differentiation assays. The cells were routinely tested for mycoplasma contamination.

### In vivo implantation assay and sample testing

The experimental procedures were approved by the ethics committee and local competent authorities (see the “ethics approval” section) (ARRIVE as a checklist as Additional files 1, 2 and 3).

*Escherichia coli*-produced recombinant BMP-2 (rhBMP-2) was obtained from commercial providers (Noricum SL, Cat.# BMP2). rhBMP-2 and hBM-MSC carrier implants were prepared as previously described [17, 18]. Briefly, Gelfoam gelatin sponges (Pfizer, Cat.# 0009-0323-01) were sectioned into 48 pieces, washed with 70% ethanol (Scharlab, Cat.# ET0003005P) and rehydrated in sterile phosphate-buffered saline (PBS) (Gibco, Cat.# 14040). hBM-MSCs were diluted in culture media at 1 × 10^6^ cells/mL, and 100 µL (1 × 10^5^ cells) of the mixture was carefully inoculated in each scaffold using a 1 mL syringe (Braun, Cat.# 9161406V) with a 25G needle (Braun, Cat.# 9186166). The cell-seeded scaffolds were transferred to polystyrene ultralow attachment 24-well plates (Corning, Cat.# 3473) and maintained under cell culture conditions for 3–5 h. Then, culture media was added, and the scaffolds were cultured for 3–7 days. Five microliters of 50 mM acetic acid (Fluka, Cat.# 27225) was added to reconstituted rhBMP-2 (Noricum, Cat.# rhBMP-2) (at a concentration of 5 µg/µL). Then, 30 µL of 2% CaCl_2_ reconstituted thrombin from human plasma (Merck, Cat.# T8885), and 30 µL of water reconstituted fibrinogen from human plasma (Merck, Cat.# F3879) were added. Clotting was allowed for 10 min in cell culture conditions before proceeding with in vivo implantation.

Ten-week-old healthy female NOD-SCID (Charles River laboratories, strain code 394) mice were used for in vivo implantation studies. All animals were housed in ventilated cages and fed a standard diet ad libitum. The animals were randomly allocated to the following groups: 1—control gelatin sponges; 2—control gelatin sponges and rhBMP-2; 3—hBM-MSC carrier gelatin sponges; and 4—hBM-MSC carrier gelatin sponges with rhBMP-2. The sample size was established at n = 3 per group and two implantation times according to the primary outcome measure (histology). A total of 24 animals were used. Anaesthesia was induced with 2.5% isoflurane and O_2_ at 2–4%. The analgesic and anti-inflammatory agent chaprofen (Zoetis, Rimadyl) (5 µg/g of animal) was administered subcutaneously. A wide section of the fur from the back was shaved. Then, the skin was sterilized twice with povidone-iodine (Mylan, Cat.# Betadine). For each scaffold implantation, a 0.5 cm incision was made in the skin located just on top of the spine of the animal. With forceps and a skin-generated incision, a pocket under the skin was made down the side of the animal. A scaffold was inserted, ensuring that it was placed deep within the pocket, and then the incision was stapled and cleaned with povidone-iodine. Anaesthesia was removed, and the animals were supervised until total recovery. The analgesic and anti-inflammatory Carprofen (5 µg/g of animal) was reconstituted in PBS and administered subcutaneously every 24 h for 3 days after surgery. An overdose of carbon dioxide (CO_2_) was used to euthanize the animals. Samples were harvested for routine histology, paraffin embedding and sectioning at 4 µm, aiming to assess the phenotype of the formed tissue and the contribution of the implanted hBM-MSCs. No exclusions were done. Sections were subjected to hematoxylin and eosin staining (Abcam, Cat.# ab245880) and alcian blue staining (Merck, Cat.# TMS-010-C) with hematoxylin counterstaining (Abcam, Cat.# ab245880). Mouse non-cross-reactive, human vimentin-specific antibody (Abcam, Cat.# ab8069), perilipin antibody (Progen, Cat.# 690156S) and DAPI (Merck, Cat.# D8417) were used for immunofluorescence staining. Optical and confocal microscopy images were obtained. The group allocation was blinded at the outcome assessment and data analysis steps.

### Alkaline phosphatase (ALP) activity testing

All cell culture assays with rhBMP-2 were performed at a final concentration of 0.5 µg/mL. The ALP activity of the cells was evaluated as previously reported [19]. For histology, naphthol AS-BI and fast red violet LB-based alkaline phosphatase staining kits were used (Merck, Cat.# 86R), and the manufacturers’ indications were followed. For quantitative data, a StemTAG™ Alkaline Phosphatase Activity Assay Kit (Cell Biolabs, Cat.# CBA-301) was used, following the manufacturer’s instructions. ALP activity data were normalized to blank medium and to total cellular protein content measured by Bradford assay (Bio-Rad, Cat.# 50000006) [4]. The resulting values were normalized to those of the controls to obtain the fold change.

### Total DNA and mitochondrial metabolic activity testing

For total deoxyribonucleic acid (DNA) analysis, cells were washed in PBS and treated with 70% methanol to kill them. Further on, Ethidium Homodimer 4 μM ethidium homodimer in PBS (Thermo Fisher, Cat.# E3599) was added. After 30 min of incubation in the dark, the data were collected (Ex 530 mm, em 645 nm) in a Biotek FL-600 plate reader. For mitochondrial metabolism activity measurement, the MTT-based CellTiter 96® Non-Radioactive Cell Proliferation Assay Kit was used (Promega, Cat.# G4000) following the manufacturer’s instructions (readout at 590 nm and 630 nm in a Biotek FL-600 plate reader).

### Quantitative real-time PCR (qPCR)

Primers for the target genes were designed using the primer-BLAST primer design tool (NIH, USA) (Additional file 3: Supplementary Table 1) and purchased from Merck. An RNeasy Plus Mini Kit (Qiagen, Cat.# 74134) was used to remove genomic DNA (gDNA) and isolate total ribonucleic acid (RNA) according to the manufacturer’s instructions. The RNA concentration was measured using a Nanodrop spectrophotometer (Thermo Fisher Scientific). RNA was reverse transcribed to cDNA and subjected to RT-PCR using a GoTaq (R) 2-Step RT-qPCR Kit (Promega Biotech Iberica, Cat.# A6010) following the manufacturer’s instructions. All the reactions were conducted in triplicate. Real-time PCR was performed using a CFX Connect Real-time PCR detection system (Bio-Rad), and differential gene expression was calculated by using the 2^−ΔΔCt^ method. The gene expression levels of the target genes were measured by normalization to the housekeeping gene *GAPDH,* exploiting control samples as calibrators. The data were normalized to the data of cells seeded on a control cell culture standard plastic surface. All the reactions were conducted in triplicate.

### RNA Sequencing

The quantity and quality of the RNAs were evaluated using a Qubit RNA HS Assay Kit (Thermo Fisher Scientific, Cat.# Q32855) and Agilent RNA 6000 Nano Chips (Agilent Technologies, Cat.# 5067-1511), respectively. Sequencing libraries were prepared following the “TruSeq Stranded mRNA Sample Preparation Guide (Part # 15031058 Rev. E)” using the “TruSeq® Stranded mRNA Library Prep” kit (Illumina, Inc., Cat. # 20020594) and the TruSeq RNA CD Index Plate (96 Indexes, 96 Samples) (Illumina, Inc., Cat. # 20019792). Starting from 700 ng of total RNA, mRNA was purified, fragmented and primed for cDNA synthesis. The cDNA first strand was synthesized with SuperScript-II Reverse Transcriptase (Thermo Fisher Scientific, Cat. # 18064-014) for 10 min at 25 °C, 15 min at 42 °C, 15 min at 70 °C and a pause at 4 °C. The cDNA second strand was synthesized with Illumina reagents at 16 °C for 1 h. Then, A-tailing and adaptor ligation were performed. Finally, enrichment of the libraries was achieved by PCR (30 s at 98 °C; 15 cycles of 10 s at 98 °C, 30 s at 60 °C, and 30 s at 72 °C; 5 min at 72 °C; and a pause at 4 °C). Afterwards, the libraries were visualized on an Agilent 2100 Bioanalyzer using an Agilent High Sensitivity DNA Kit (Agilent Technologies, Cat. # 5067-4626) and quantified using a Qubit dsDNA HS DNA Kit (Thermo Fisher Scientific, Cat. # Q32854). Libraries were sequenced on a NovaSeq 6000 (Illumina, Inc.), generating 101 bp paired-end reads.

### RNA-sequencing data analysis

RNA-sequencing data analysis was conducted as previously described [20]. We used HISAT2 [21] to align the RNA-seq reads to the human reference genome hg38 and Cufflinks [22] to annotate them. We calculated the counts of aligned reads to each gene with HTSeq [23]. We merged the transcriptomics data into a single text file and used it in the downstream analysis in MATLAB. We equalized the data and stabilized them through the log_2_ transform of the data plus one; calculated the average values for each group of replicates; selected the differentially expressed genes (DEGs) whose absolute difference in mean values between the two groups was less than the selection threshold θ_DEG_ = 1 of fold change in the log_2_ scale; and selected the statistically significant DEGs using Student’s t test with a significance threshold of 0.05. DEG sets were subjected to Gene Ontology (GO) enrichment analysis (http://geneontology.org). Additionally, we performed GSEA (http://software.broadinstitute.org/gsea/msigdb). The scatter plots, principal component analysis (PCA), GO analysis and GSEA were performed using in-house functions developed in MATLAB (MathWorks).

### Differentiation media and cell staining

Osteogenic differentiation was induced with the Osteogenic Differentiation Medium Bullet Kit (Lonza, Cat.# PT-3002). Adipogenic differentiation was induced with adipogenic induction medium (Lonza, Cat.# PT-3102B). For chondrogenic differentiation assays, cells were deposited in pellets (5 × 10^5^ cells/pellet), and differentiation was induced with chondrogenic differentiation medium (Stem Cell Tech, Cat.# 5455). Dexamethasone (10 nM; Merck, Cat.# D4902), B-glycerol phosphate (BGP) at different concentrations (Merck, Cat.# G9422) and ascorbic acid (200 μM; Merck, Cat.# A4544) were also used in differentiation assays. When needed, the media were supplemented with 0.5 µg/mL rhBMP-2.

Alizarin red staining was used to stain the extracellular matrix calcium deposits red. In this sense, the cells were fixed in 70% ethanol, stained with alizarin red S (Merck, Cat.# TMS-008-C) and washed with water. Oil Red O staining was used to stain the adipose droplets red. In brief, 6 ml of Oil Red O solution (Merck, Cat.# O1391) was diluted with 4 ml of distilled water and filtered afterwards to remove the precipitants. The cells were washed with PBS and fixed with 4% formalin at room temperature. Thereafter, the cells were washed with PBS and isopropanol (60%). Oil Red O working solution was added to the samples for 1 min, after which the samples were washed with PBS to remove the excess dye. Alcian blue staining was used to stain the cartilage glycosaminoglycans (GAGs) blue. In this case, cell pellets were processed for paraffin embedding and sectioning prior to histology staining with alcian blue (Merck, Cat.# TMS-010-C) and counterstaining with hematoxylin (Abcam, Cat.# ab245880). All the samples were observed by an optical microscope (Motic, Cat.# BA310).

### Statistics

All the data were plotted and statistically analysed using GraphPad Prism software (La Jolla, CA, USA). In all the cases, the normality of the data was verified using the Shapiro–Wilk test, and a normal QQ plot was generated. The data that passed the normality test (α = .05) were statistically analysed using parametric tests (ordinary one-way ANOVA) and Tukey’s multiple comparisons tests. The data are presented as the means±SDs, and the values for each individual point are provided in the plots. Additionally, the *n* number of each assay is provided in the figure legends. *P* values less than 0.05 were considered to indicate statistical significance (ns *p* > 0.05; *p < 0.05; **p < 0.01; ***p < 0.001).

## Results

### In vivo, rhBMP2 induces multilineage differentiation in hBM-MSCs

**Fig. 1:**
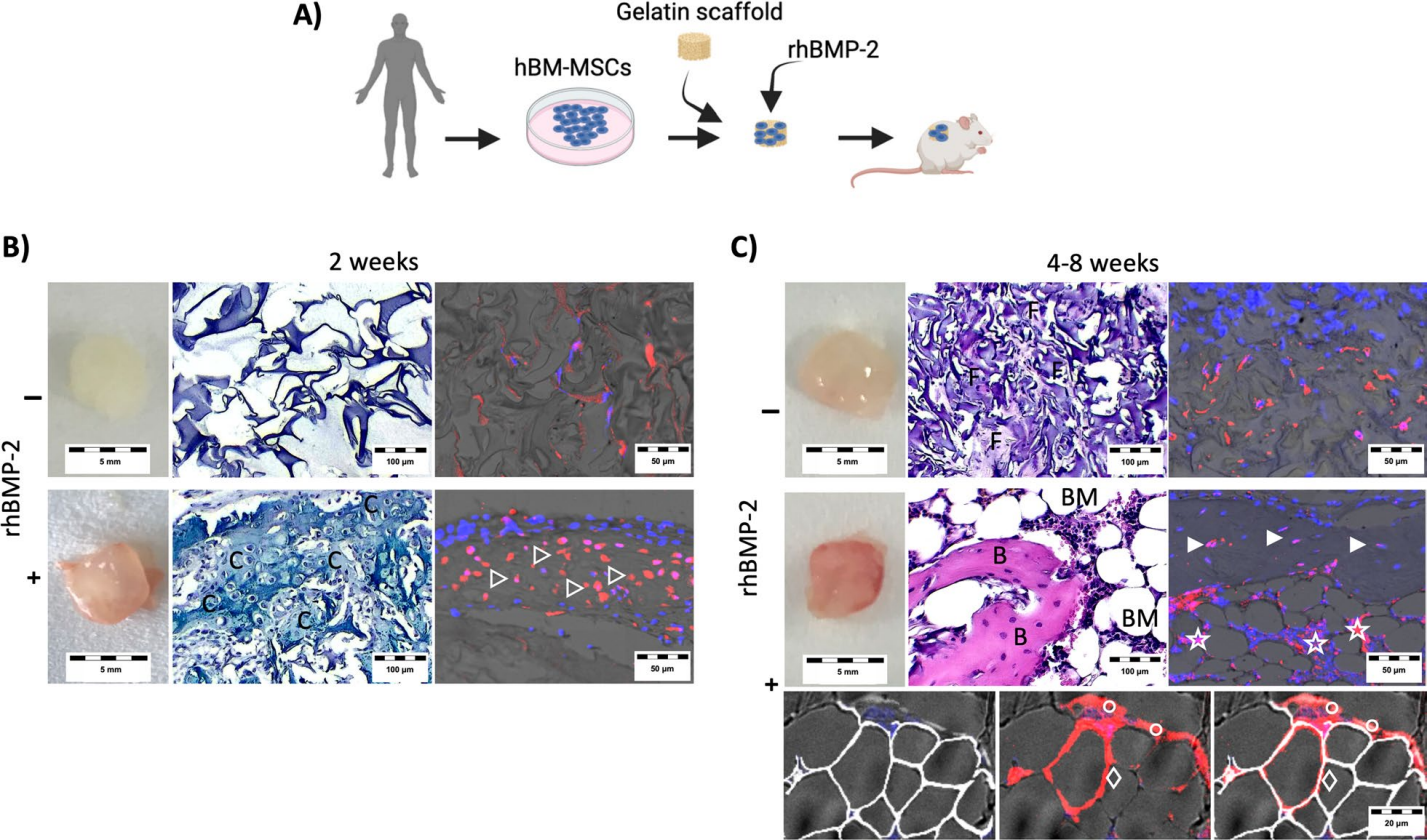
rhBMP2 induces in vivo multilineage differentiation in hBM-MSCs. **A** Schematic of hBM-MSC loading on a gelatin carrier scaffold, followed by rhBMP-2 addition and implantation in mouse subcutaneous tissue. **B**, **C** Gross morphology and histology of harvested samples. **B** Samples 2 weeks after implantation. The gross morphology of the sections stained with alcian blue (C, chondrocyte tissue). Immunostaining with nuclei counterstaining (blue) and a mouse non-cross-reactive, human-specific vimentin antibody were used to locate the implanted human cells (red arrowheads indicate chondrocytes). **C** Samples after 4–8 weeks of implantation. Gross morphology of H&E-stained sections (F, fibrous tissue; B, bone tissue; BM, bone marrow area). Immunostaining revealed nuclear counterstaining (blue), Perilipin adipocyte staining (white) and human vimentin-positive cells (red: arrowheads, osteocytes; stars, bone marrow stromal cells; diamonds, adipocytes; circles, bone lining osteoblasts). The data shown correspond to donor D24 (n = 3 per condition). The data were confirmed in donors D19 and D23

Our first aim was to test the ability of rhBMP-2 to promote de novo bone formation and the multilineage differentiation of hBM-MSCs. A gelatin hydrogel was used as a carrier for rhBMP-2 and hBM-MSCs at passages 2–4 to establish a xenograft in immunocompromised mice (Fig. 1A). After 2 weeks (Fig. 1B), the harvested samples were solid, and the histology confirmed cartilage formation, as indicated by the observation of chondrocyte cell morphology and positive staining for the cartilage extracellular matrix (Alcian blue). Moreover, immunostaining indicated that the implanted hBM-MSCs were incorporated into the tissue as chondrocytes. The control samples generated from hBM-MSCs, but not from rhBMP-2, showed no tissue formation in the implanted material, and the implanted hBM-MSCs showed a fibroblastic phenotype inside the implants. At 4–8 weeks (Fig. 1C), the control samples did not exhibit any specific tissue formation, and the implanted hBM-MSCs show fibroblasts phenotype inside the implant. However, the rhBMP-2 carrier implants were solid structures with evident vascularization at gross morphology. Histology revealed mature tissue formation in these samples, with bone and adipocytic-hematopoietic vascularized bone marrow tissue. Immunostaining of these samples revealed that the implanted hBM-MSCs differentiated into osteoblasts, osteocytes inside the bone tissue, adipocytes and stromal cells in the bone marrow area.

These data indicate that rhBMP-2 triggers physiological and sequential molecular mechanisms and events related to bone formation via endochondral ossification, from initial cartilage callus formation to the final emergence of bone tissue with vascularized and hematopoietic mature bone marrow. Moreover, the hBM-MSCs present at the implant site participate in all these events and are differentiated into all mesenchymal phenotypes observed in the ossicles, such as chondrocytes, osteoblasts, osteocytes and adipocytes, indicating their multilineage differentiation ability. Therefore, the rhBMP-2 used in this study induces in vivo bone formation and in vivo multilineage differentiation in hBM-MSCs.

### In vitro, rhBMP-2-treated hBM-MSCs exhibited differential marker expression in the short-term but no differentiation in long-term culture

**Fig. 2:**
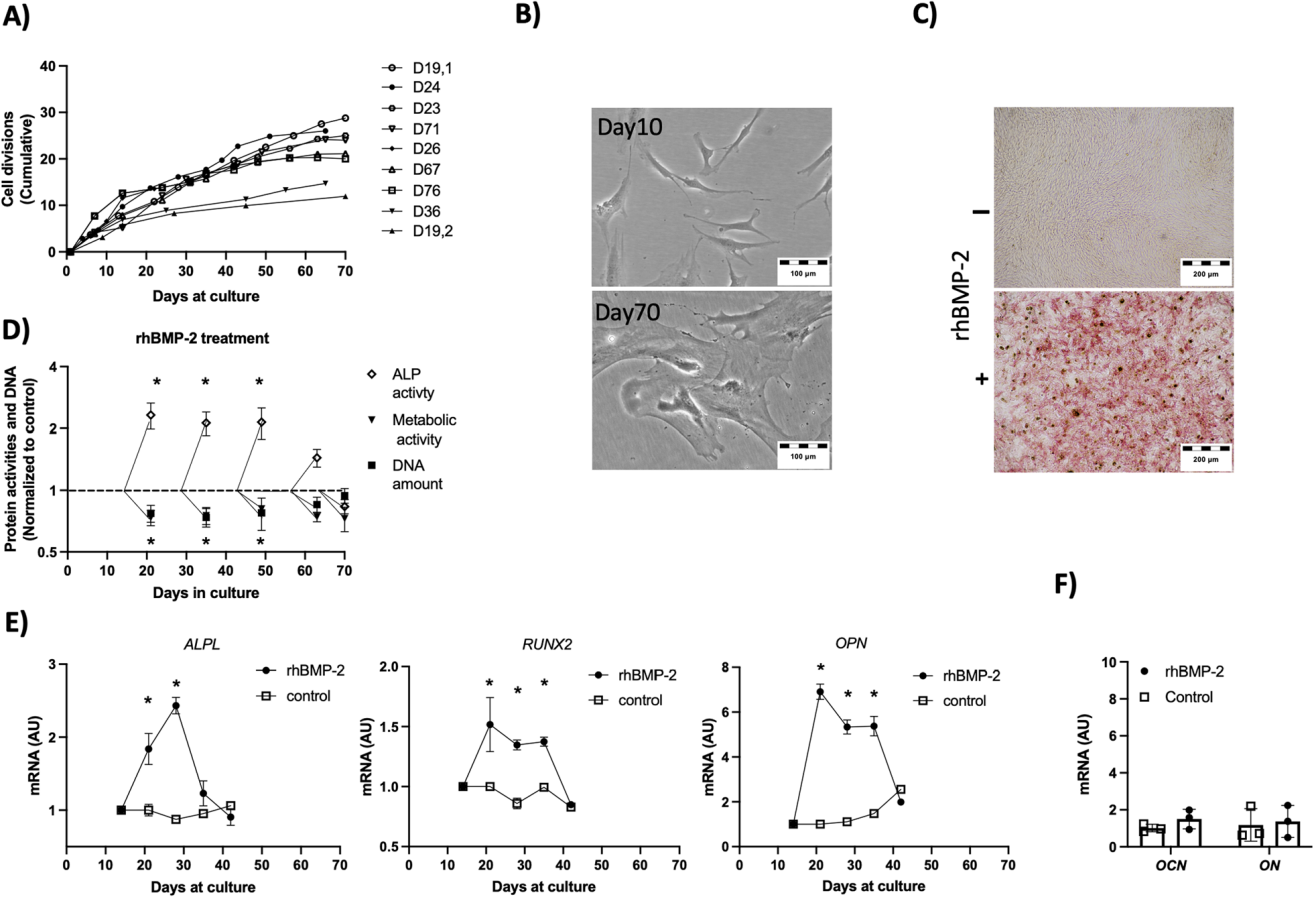
In vitro-cultured hBM-MSCs respond to rhBMP-2 treatment. **A** In vitro cell culture growth kinetics of hBM-MSCs. (Cells from 9 donors were included). **B** hBM-MSCs appearance at early cell culture (10 days) and late cell culture (70 Days) points. **C** ALP staining (red) of early hBM-MSCs cultured for 7 days with rhBMP-2. **D** Multiple parameters of hBM-MSCs treated for 7 days at different time points. The data are presented relative to the number of days of culture for the hBM-MSCs. Colorimetric ALP activity was measured as an indicator of an early differentiation marker, ethidium homodimer DNA was used as an indicator of a proliferation marker, and MTT mitochondrial activity was used as an indicator of cell metabolic activity. All the data are presented normalized to their own control on the day of the start of the assay. The first assay was started on day 14 after starting the cell culture. n = 3 for each experimental point. The data shown correspond to donor D19. The induction of ALP was confirmed in donors D23, D24, D26, and D76. **E** Kinetics of selected genes in response to continuous rhBMP-2 treatment for 28 days. Note that the assay started on day 14 after the cell culture started. n = 3 for each experimental point. The data shown correspond to donor D19. The data were confirmed in donors D23, D24, and D26. **F** Gene expression profile of selected genes after 28 days of rhBMP-2 treatment. n = 3 for each experimental point. The data shown correspond to donor D19. The data were confirmed in donors D23, D24, and D26. *ALPL*, alkaline phosphatase biomineralization associated; *RUNX2*, RUNX Family Transcription Factor 2; *OPN*, osteopontin; *OCN*, osteocalcin; *ON*, osteonectin (stars) indicate statistically significant differences (*p* < 0.01)

hBM-MSCs from all different donors were expanded to determine their cell culture proliferation potential (Fig. 2A). Interdonor variability was also observed regarding proliferation kinetics, remarkably for two donors, which exhibited lower proliferation avility. Morphology was similar for the cells of all donors; mostly fibroblastic spindle-shaped cells were observed at early culture periods, and large and flat morphology at later passages (Fig. 2B). At early culture periods, cells from all donors exhibited increased ALP expression, as assessed by ALP activity staining after 7 days of treatment with rhBMP-2 (Fig. 2C). At this point, hBM-MSC donor batches that did not proliferate properly (Donors D36 and D19,2) and the hBM-MSC donor batches that did not show increased ALP activity after rhBMP-2 treatment (Donors D71 and D67) were excluded from further studies.

After different culture periods, the hBM-MSCs were treated with rhBMP-2 for 7 days (Fig. 2D). Assays performed with cells in short-medium cell culture periods revealed increased ALP activity, reduced mitochondrial metabolic activity, as measured by the MTT assay, and a decreased DNA concentration, as measured by the ethidium homodimer assay. However, assays performed with cells previously cultured for more than 60 days did not reveal a response to rhBMP-2 treatment.

After 15 days, the cells were treated with rhBMP-2 for the following 28 days (Fig. 2E). The increase in the expression of several specific genes, such as *ALPL*, *RUNX2* and *OPN,* was detected every 7 days at early time points. At the late time point (28 days), the expression of these genes was similar to that in the control nontreated hBM-MSCs. Additionally, the expression of the osteogenic markers *OCN* and *ON* did not differ from those of the controls.

**Fig. 3:**
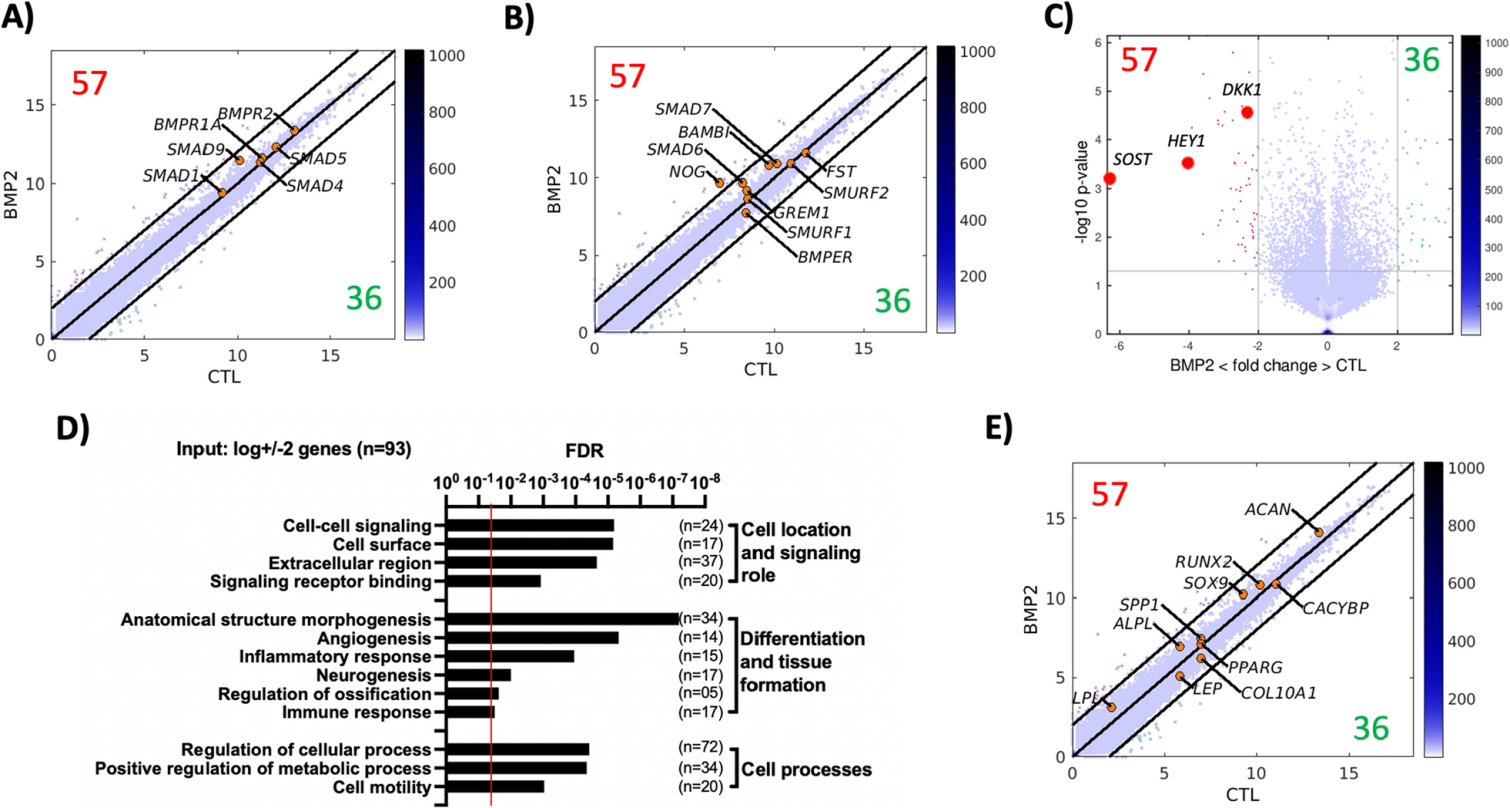
Long-term rhBMP-2 treatment of hBM-MSCs does not induce global gene expression rearrangement. The control and rh-BMP-2-treated hBM-MSCs were cultured for 28 days. **A**, **B** Scatter plot of gene expression (axes provided on a logarithmic scale) showing selected genes. Lines indicate the limits of differential overexpression or downregulation. The number 57 indicates the genes differentially overexpressed in the rhBMP-2 treatment group. The number 36 indicates the genes downregulated in the rhBMP-2 treatment group. **A** The key genes involved in the BMP-2 signalling pathway (none of which were differentially expressed). **B** The main inhibitors of the BMP signalling pathway (noggin (NOG) was the only one differentially overexpressed in the rhBMP-2 treatment group). **C** Volcano plot of gene expression data showing the overexpression of three rhBMP-2 signalling inhibitor genes related to WNT/β-catenin signalling. **D** GO terms obtained in the GO analysis of all the differentially expressed genes (Input: log ± 2 genes. n = 93). **E** Scatter plot of gene expression showing the main osteogenic, adipogenic and chondrogenic differentiation markers. (None of the genes were differentially expressed). Assays were conducted using cells from donor D24. Independent replicate samples (n = 3 per condition) are shown in Additional file 1: Fig. 1

The assay was repeated to perform a global gene expression study of control samples *and* 28-day rhBMP-2-treated cells (Fig. 3). The principal component analysis showed good separation between the triplicates of those two groups, indicating differences between groups at the global transcriptomics level (Additional file 1: Fig. 1). The mean transcriptomic expression values are represented as a scatter plot of gene expression to visualize the relative expression of each gene under each condition. The results indicated that the main genes related to the BMP pathway were relatively highly expressed in both the control and rhBMP-2-treated cells but with no differential expression related to the treatment groups (Fig. 3B). The main BMP signalling inhibitors were also located within the scatter plot, and they were expressed at similar levels in both groups, except for Noggin (*NOG*), which was overexpressed in the rhBMP-2-treated samples. The Gene Ontology (GO) enrichment analysis based on gene set enrichment analysis (GSEA) revealed that WNT signalling was a route altered by rhBMP-2 treatment (Additional file 1: Fig. 1); therefore, we identified BMP signalling inhibitors related to WNT signalling (*SOST, DKK1* and *HEY1*), and these genes are indicated in the volcano plot of the transcriptomic data (Fig. 3C). Furthermore, a GO analysis was performed on the 96 genes differentially expressed between the control and BMP2 conditions (Fig. 3D). In terms of differentiation, they were related to terms broadly related to regeneration, such as morphogenesis and angiogenesis, but not to specific differentiation patterns, such as osteogenesis, adipogenesis or chondrogenesis. The main osteogenic, chondrogenic and adipogenic differentiation markers were identified in a scatter plot (Fig. 3E), which shows that they were expressed but not differentially overexpressed or downregulated compared to those in the control group.

### In vitro, rhBMP-2 does not induce terminal differentiation in hBM-MSCs but synergizes with multiple differentiation media to achieve this effect

**Fig. 4:**
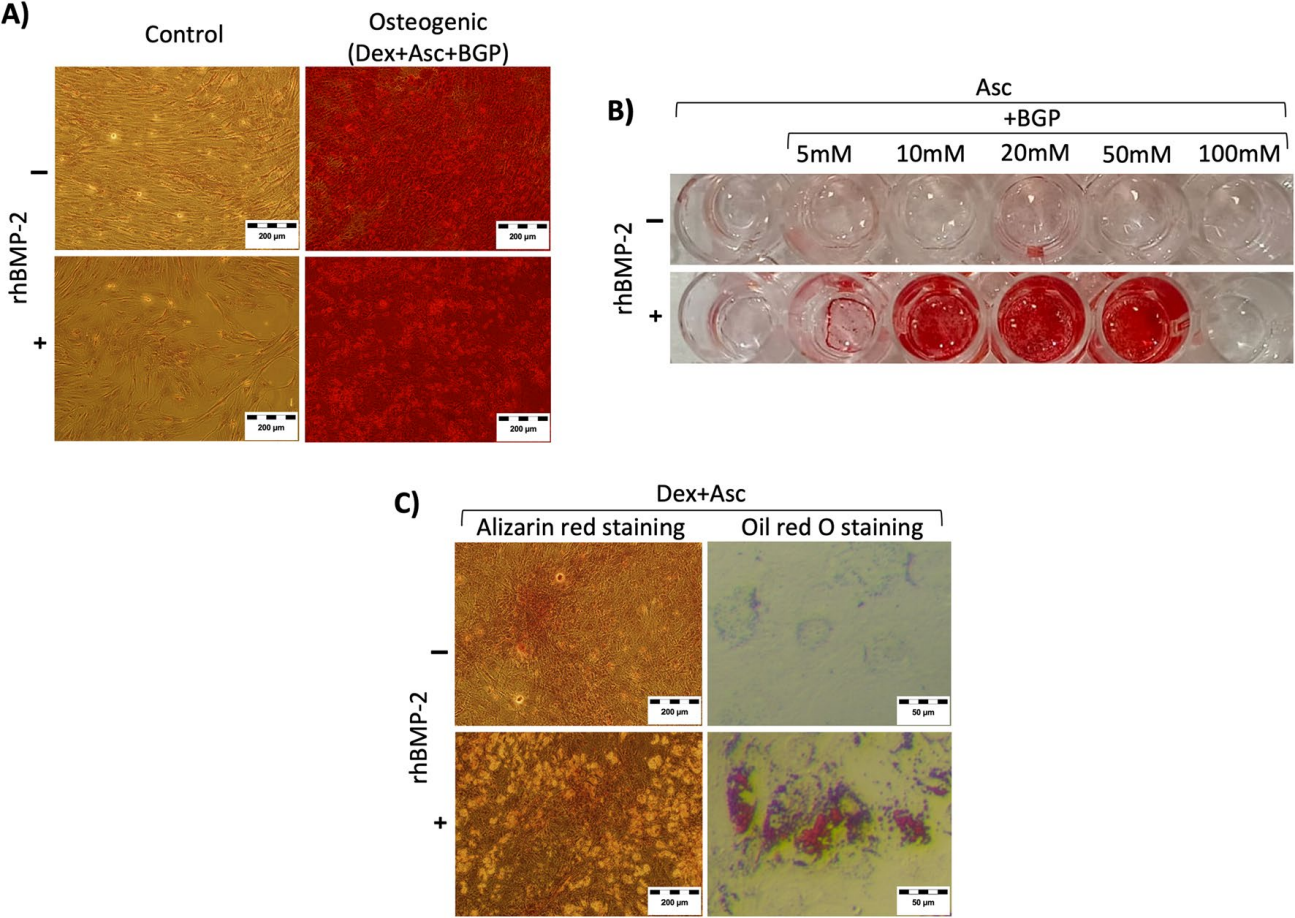
Effect of rhBMP-2 treatment in combination with the osteogenic differentiation media components. Images of cells stained after 28 days of incubation. **A** Alizarin red staining images of cells incubated in control or complete osteogenic media with or without rhBMP-2. Red staining indicates calcium deposition in the extracellular matrix. **B** Alizarin red staining images of cells incubated in control media supplemented with ascorbic acid and different concentrations of B-glycerophosphate, with or without rhBMP-2. Red staining indicates calcium deposition in the extracellular matrix. **C** Alizarin red staining and Oil red O staining images of cells incubated in control media supplemented with dexamethasone and ascorbic acid and with or without rhBMP-2. Oil red O staining indicated adipose droplets. (Dex, dexamethasone; Asc, ascorbic acid; BGP, B-glycerophosphate). n = 3 for each experiment. The data shown correspond to donors D19, D23, D24 and D26

rhBMP-2 was used in combination with other chemical reagents which has well-known roles in the induction of osteogenesis (Fig. 4). First, we tested the use of rhBMP-2 as a supplement within complete osteogenic differentiation media and obtained samples for calcium deposit staining at 14, 21 and 28 days, which revealed that rhBMP-2 supplementation accelerated calcium deposition (Fig. 4A and Additional file 2: Fig. 2).

Then, rhBMP-2 was used in combination with each one of the specific components which trigger osteogenic differentiation in vitro, aiming to determine whether it could replace any of the other components. At 28 days, the combination of rhBMP-2 with B-glycerophosphate (BGP) and ascorbic acid (Asc) yielded spots of calcium deposition only within a range of B-glycerophosphate (BGP) concentrations (Fig. 4B). On the other hand, the combination of rhBMP-2 with dexamethasone (Dex) induced an unexpected adipogenic differentiation, suggesting that rhBMP-2 plays a key role in promoting adipocyte differentiation (Fig. 4C).

**Fig. 5:**
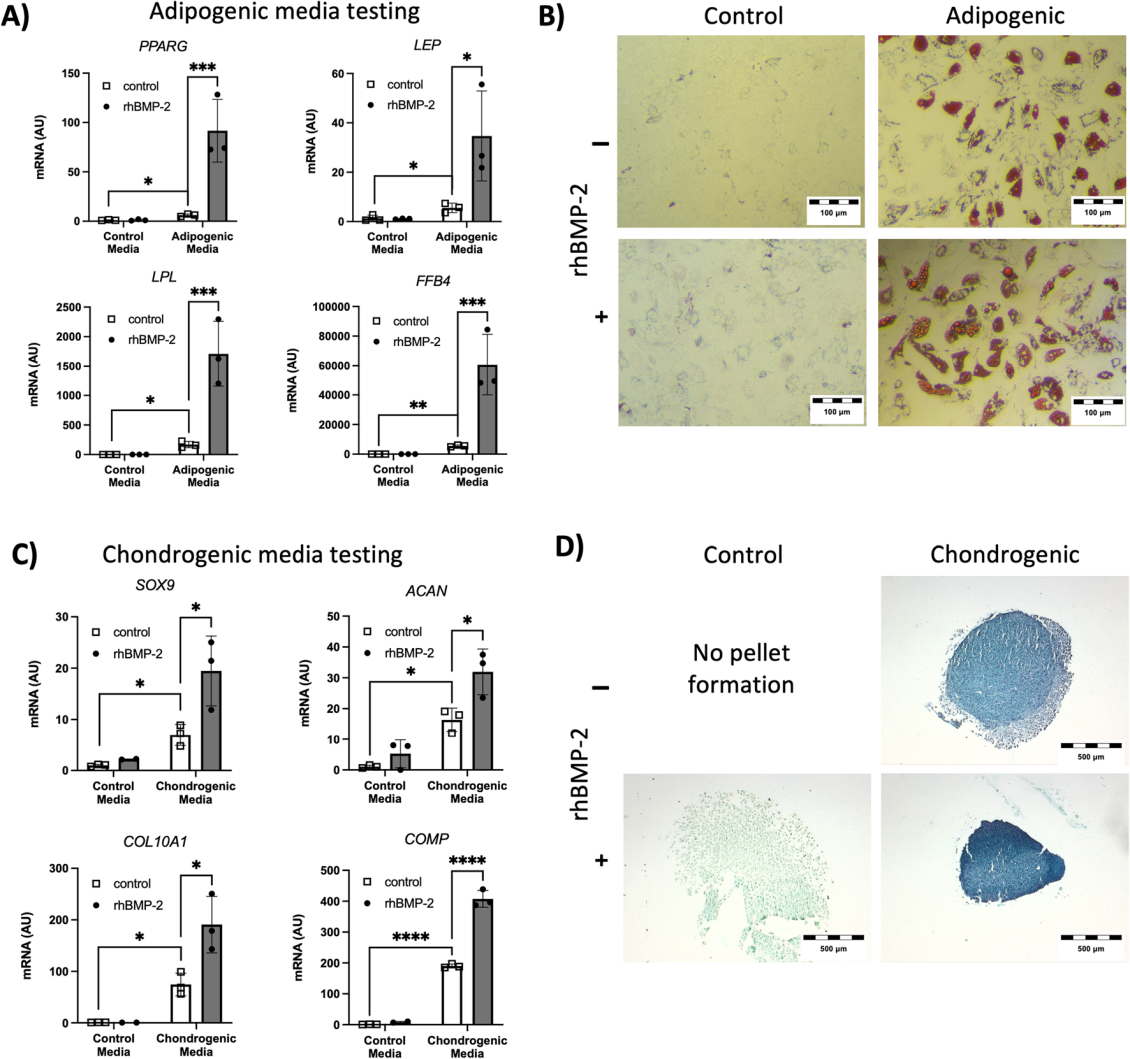
rhBMP-2 synergically potentiates the adipogenic and chondrogenic differentiation of hBM-MSCs. **A** Gene expression data for four representative adipogenic markers. The cells were cultured in control media or adipogenic media supplemented with or without rhBMP-2. **B** Oil red O staining images of cells incubated in control media or adipogenic media with or without supplementation with rhBMP-2. Red staining indicates adipose droplets. **C** Gene expression data for four representative chondrogenic markers. The cells were cultured as pellet cultures in control media or chondrogenic media, with or without supplementation of rhBMP-2. **D** Alcian blue staining images of pelleted cultured cells incubated in control media or chondrogenic media with or without supplementation with rhBMP-2. Blue staining indicates the presence of glycosaminoglycans (GAGs). (Gene expression data were obtained after 10 days of treatment. Histology obtained after 28 days of treatment). n = 3 for each experiment and experimental point. The data shown correspond to donors D23, D24 and D26 and were confirmed on D19

Therefore, complete adipogenic media was supplemented with rhBMP-2 to determine any potential benefits on this differentiation (Fig. 5A, B). After 10 days, rhBMP-2-supplemented adipogenic medium-treated hBM-MSCs presented increased levels of multiple adipogenic markers, while this effect was not observed when the control medium was tested (Fig. 5A). At 28 days, terminal differentiation was observed in both adipogenic media-treated hBM-MSC groups, while the amount and size of the adipose droplets was bigger in the rhBMP-2-supplemented group (Fig. 5B).

Similarly, chondrogenic differentiation media and hBM-MSC pellet cultures were used to determine the effect of rhBMP-2 supplementation on chondrogenic differentiation (Fig. 5C, D). After 10 days, chondrogenic-specific markers were upregulated in the rhBMP-2-supplemented chondrogenic media-treated cells but not in the control cells or in the rhBMP-2-supplemented control media-treated cells (Fig. 5C). At 28 days, histology data indicate pellet culture was not maintained in control media group, while the increased alcian blue specific staining intensity evidenced increased levels of glicosaminoglicans (GAGs) in rhBMP-2 supplemented chondrogenic media treated hBM-MSCs, compared to only chondrogenic media samples, which also show positive alcian blue GAGs specific staining (Fig. 5D). Treatment with control media supplemented with rhBMP-2 did not induce the expression of GAGs but did induce long-term pellet formation, suggesting the expression of extracellular matrix components in this group.

## Discussion

In previous studies, we reported multiple in vivo research models based on xenotransplantation of rhBMP-2 and/or hBM-MSCs that were useful for 1-inducing bone formation, 2-studying the differentiation ability of hBM-MSCs, and 3-creating humanized bone marrow niches in mice [4, 8, 17, 18, 24–29]. Human hBM-MSCs exhibit interdonor variability, especially in terms of proliferation and differentiation potential. In a previous report, we showed that in vitro hBM-MSC multilineage differentiation does not always correlate with in vivo outcomes; therefore, it was suggested that both testing methods are required to determine the multilineage differentiation potential of each type of donor-specific hBM-MSC [8].

The literature indicates that cell-carrier material properties are major variables that affect implanted cell behaviour [30–32]. In bone tissue context, the main materials used are those mimicking the major structural components of the bone extracellular matrix, such as ceramics and collagen-based materials. Ceramics provide a calcium source for a better biomineralization, while collagens and its derivatives, as gelatin, provide proper extracellular matrix to support bone tissue formation [33]. Previously, we used tricalcium phosphate (TCP)-based ceramic carrier materials, and the approach yielded cartilage and bone formation and multilineage differentiation of implanted hBM-MSCs at 2 weeks after implantation [8]. Here, a gelatin hydrogel was used as a carrier for rhBMP-2 and hBM-MSCs, similar to what we previously tested for the creation of humanized bone marrow niches in mice [4, 8, 17, 18, 24–29]. This approach yields the formation of mature bone tissue, in which the implanted hBM-MSCs can be observed as chondrocytes, osteoblasts, osteocytes or adipocytes depending on the sample harvesting time (Fig. 1). However, we observed that, in a gelatin carrier material, the bone formation process is slower than that in the assays previously performed with ceramic carriers. In the Gelatin carrier, hBM-MSCs differentiated only to the cartilage stage of endochondral ossification at 2 weeks after implantation. This is surely related to the absence of local calcium and phosphate availability provided by the ceramic implantation material [17, 18]. Then, 4–8 weeks are required to achieve mature bone formation with the gelatin carrier material, rhBMP-2 or other cells, in which hBM-MSCs are osteoblasts, osteocytes and adipocytes within the newly formed ectopic bone and bone marrow tissue, indicating their multilineage differentiation ability in a bone formation environment. These assays revealed that the rhBMP-2 and hBM-MSCs used were indeed able to induce and participate in the in vivo mature bone formation machinery, including differentiation into related multiple mesenchymal phenotypes.

rhBMP-2 and hBM-MSCs were tested in an in vitro cell culture context to determine the ability of rhBMP-2 to induce multiple differentiation phenotypes in hBM-MSCs. hBM-MSCs are primary cells with a limited lifespan; therefore, we first tested the effect of rhBMP-2 treatment at different time points during hBM-MSC culture (Fig. 2). When cells were tested at short passages, rhBMP-2 induces the expression of ALP and other well-known early osteogenic markers. Additionally, cells exhibit a reduction in proliferation, which is in accordance with the potentiation of differentiation processes. When cells were tested after long culture periods, hBM-MSCs failed to respond to rhBMP-2 treatment in those terms, and our assumption is that it was because in vitro cell aging and senescence processes had already started, impeding cell differentiation [34]. Interestingly, rhBMP-2 treatment was repeated in short-passage cells, but after a longer treatment period, an initial increase in the expression of specific markers was observed, but a progressive decrease in the expression of the assayed markers was observed. We previously reported a global change in the gene expression profile of mesenchymal cells treated with rhBMP-2 during a short period of time [4]. However, the data presented here indicated a loss of the effect of rhBMP-2 in long-term cell culture. A global transcriptomics study (Fig. 3) confirmed that only 96 genes were differentially expressed after 28 days of treatment with rhBMP-2 in hBM-MSCs compared to untreated cells. The data indicate that all the main proteins involved in the BMP pathway were expressed in the tested cells and that only some BMP pathway signalling inhibitors were upregulated. Interestingly, the main inhibitors observed were those related to the WNT signalling pathway. BMP signalling generates negative feedback loops, and it has been previously reported that *DKK1*, *HEY-1* and *SOST*, which are inhibitors of WNT signalling, are indeed downstream targets of BMP signalling and negative regulators of bone formation [35–39]. Therefore, the transcriptomic data obtained after 28 days of rhBMP-2 treatment, in the absence of further signals, are consistent with the activation of negative feedback loops involving WNT signalling inhibitors that are able to suppress the effects of rhBMP-2 treatment during long-term in vitro cell culture periods.

Taken together, our data apparently differ from those of other previous studies reporting that rhBMP-2 alone can induce terminal osteogenic differentiation of hBM-MSCs in an in vitro cell culture context [40–45]. A detailed analysis of the experimental section of those studies showed that rhBMP-2 was indeed used as a supplement to osteogenic differentiation media and not as a unique differentiation factor. Consequently, we tested the ability of rhBMP-2 to synergize with osteogenic differentiation media components.

The osteogenic media is composed of 3 main components: dexamethasone (usually at 10 nM), ascorbic acid (Vitamin C, usually at 200 μM) and B-glycerophosphate (usually at 10 mM). Ascorbic acid (Asc) is required to potentiate collagen type 1 secretion and therefore proper extracellular matrix creation [10]. B-Glycerophosphate (BGP) is used as the source of the phosphate needed for the formation of hydroxyapatite calcium deposits in the extracellular matrix, and the phosphate provided to the system is also considered an intracellular signalling molecule able to regulate the expression of osteogenic genes [10]. On the other hand, dexamethasone (Dex) is a glucocorticoid, and its in vitro cell culture efficacy varies depending on the target cell, time, and concentration. In the hBM-MSC context, Dex prevents apoptosis and triggers MSC expansion [46], while it is used at different optimized doses not only for osteogenic (10 nM) but also for chondrogenic (100 nM) and adipogenic (1000 nM) differentiation media formulations [10, 12, 47]. Interestingly, BGP, Dex and Asc, all of them, have the potential to revert the previously mentioned negative feedback loops involving WNT signalling inhibitors, because all of them activate WNT signaling to induce osteogenesis. Dex induces WNT/β-catenin signaling-dependent Runx2 expression at osteogenesis [48]; BGP activates canonical and non-canonical WNT signalling [49], and; Asc activates osteogenesis via Wnt/β-Catenin/ATF4 Signaling Pathways [50].

We observed that, indeed, rhBMP-2-supplemented osteogenic media resulted in earlier calcium deposition (Additional file 2: Fig. 2), suggesting that the synergistic effect of osteogenic media and rhBMP-2 was the effect observed in previous reports [40–45]. However, After 28 days, the supplementation of complete differentiation media with rhBMP-2 did not provide any additional benefits in terms of calcium deposition (Fig. 4A).

At this point, we wondered which of the specific components was involved in the interaction of rhBMP-2 and whether rhBMP-2 could replace any of the other components of the osteogenic media. First, we checked whether rhBMP-2 interacts with the BGP and can replace Dex (Fig. 4B). In our study, when Asc + BGP was used, hBM-MSCs did not undergo mineralization, indicating that additional signalling was required for osteogenic differentiation (Fig. 4B). Interestingly, when Asc + BGP was supplemented with rhBMP-2, calcification was observed at concentrations ranging from 5 to 50 mM (Fig. 4B), while higher concentrations of BGP impeded rhBMP-2-induced mineralization. On the basis of these data, we infer that the presence of a phosphate source (BGP) synergizes with the rhBMP-2 signal to promote in vitro hBM-MSC terminal osteogenic differentiation, suggesting that in vitro rhBMP-2 can be used as a substitute for dexamethasone for osteogenic differentiation.

Then, we checked whether rhBMP-2 interacts with Dex and can replace BGP. When Dex + ASC was used, hBM-MSCs did not undergo mineralization, indicating that BGP or any other phosphate source is required for in vitro mineralization (Fig. 4C). However, rhBMP-2 supplementation in combination with Dex + ASC triggered unexpected adipogenic differentiation (Fig. 4C). In this case, we can conclude that rhBMP-2 synergizes with Dex signalling to induce adipogenic differentiation but not osteogenic differentiation. Therefore, as an unexpected result of this study of the potential synergistic effects of rhBMP-2 and osteogenic media components, we concluded that, in combination with dexamethasone, rhBMP-2 may serve as a substitute for insulin and IBMX for adipogenic differentiation in vitro [12] but not for osteogenic differentiation.

With these data, we wondered whether rhBMP-2 could potentiate multiple differentiation processes simultaneously appart from osteogenesis, or at least those observed during rhBMP-2-induced in vivo mature bone formation, as it shown in Fig. 1; chondrogenic and adipogenic differentiation. First, complete adipogenic media was supplemented with rhBMP-2, and the data showed improved adipogenesis in the short- and long-term studies (Fig. 5A-B). These data confirmed the interactions between Dex and rhBMP-2 in inducing adipogenesis, while the synergistic effect of rhBMP-2 on stimulating adipogenesis has been reported previously in other cell contexts, especially in preadipocyte- or human adipose tissue-derived cells [15, 51, 52]. Similarly, rhBMP-2 supplementation in chondrogenic media also improved short- and long-term chondrogenic differentiation (Fig. 5C, D). We found that this synergistic effect on chondrogenic differentiation was previously reported in the ATDC5 mouse prechondrogenic cell line [53] but not in the context of primary hBM-MSCs.

Altogether, in vitro results indicate that rhBMP-2 potentiates not only osteogenic but also adipogenic and chondrogenic differentiation by interacting with specific components of the media, such as BGP and/or Dex. Therefore, depending on which are the components of the environment, rhBMP-2 could synergize with them to drive the trilineage differentiation potential of hBM-MSCs.

## Conclusions

rhBMP-2 is an osteoinductive factor able to induce the endochondral ossification process in vivo, yielding mature bone and bone marrow formation. This sequential process is influenced by host signals and cells and can induce multilineage differentiation of implanted hBM-MSCs. In vitro, short-term hBM-MSCs treated with rhBMP-2 expressed early osteogenic markers and multiple functional modifications. However, continuous exposure to rhBMP-2 signalling does not induce terminal differentiation in vitro due to negative feedback loops involving osteogenesis inhibitors linked to the WNT signalling pathway.

When rhBMP-2 is combined with other signals, osteogenic, adipogenic and chondrogenic differentiations are improved, all of which are phenotypes present during the in vivo bone formation process in hBM-MSC xenografts.

**Fig. 6:**
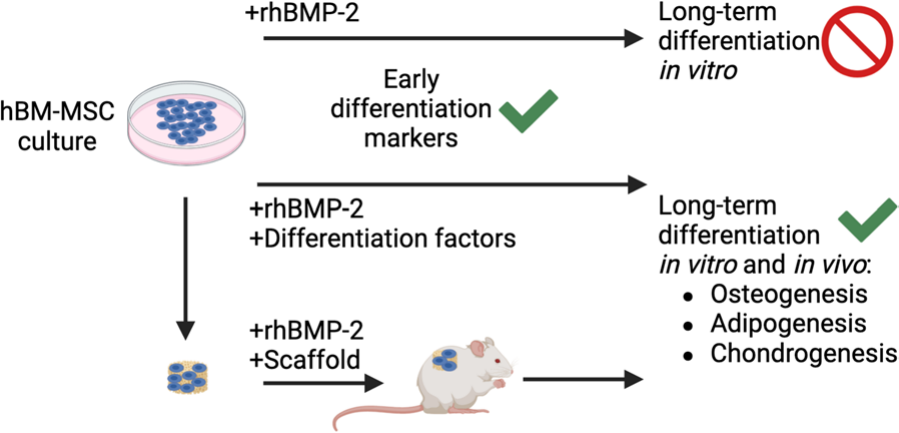
Schematic representation of the ability of hBM-MSCs to differentiate in response to rhBMP-2. In vitro, rhBMP-2 treatment induces early osteogenic-chondrogenic marker expression in short-term cultures, while long-term culture fails to induce terminal differentiation features. rhBMP-2 supplementation in osteogenic, adipogenic or chondrogenic differentiation media yields a synergistic effect, potentiating a terminally differentiated phenotype in long-term culture. In vivo, hBM-MSCs implanted in a gelatin carrier scaffold undergo multilineage differentiation due synergy between rhBMP-2 and in vivo bone formation signals

Taken together, these findings suggest that rhBMP-2 alone is unable to induce hBM-MSC terminal differentiation but synergizes with other signals to potentiate multiple differentiation phenotypes (Fig. 6). When rhBMP-2 and hBM-MSCs are used for translational bone formation applications, they have to be considered regenerative agents that act in combination with other signals and trigger differentiation depending on the signalling environment to eventually achieve proper mature bone and bone marrow formation with differentiation into multiple cell phenotypes.

### Supplementary Information

**Additional file 1: Figure 1**. **A** Principal component analysis of RNA-seq samples. Blue, control triplicate samples; red, rhBMP-2-treated triplicate samples. The two groups were separated. All of them were from donor D24 hBM-MSCs. **B** Specific gene set enrichment terms obtained in the GSEA. The WNT and B-catenin signalling terms are related to the BMP signalling inhibitor genes shown in Fig. 4. Specifically, HEY1 and DKK1 were the top 2 genes upregulated in the rhBMP-2-treated cells in this gene subset. Note that the normalized enrichment score (NES) is not far from 1, indicating that the biological meaning is subtle. Therefore, all specific gene sets identified by GSEA with very low *P* values had high false discovery rate (FDR) indices.

**Additional file 2: Figure 2**. rhBMP-2 supplementation in osteogenic media induces faster calcium deposition during in vitro osteogenic differentiation. Alizarin red staining images of cells incubated for 7, 14 or 21 days in complete osteogenic or control media supplemented with or without rhBMP-2. Red staining indicates calcium deposition in the extracellular matrix. The data shown correspond to D19 hBM-MSCs.

**Additional file 3**. Table of primers used in this study.

## Data Availability

The RNA-seq data reported in this study are accessible through the GEO database under the accession number GSE221289.

## Abbreviations

*ALPL*: Alkaline Phosphatase Biomineralization-Associated gene
Asc: Ascorbic acid
BGP: B-glycerol phosphate
DEGs: Differentially expressed genes
Dex: Dexamethasone
*DKK1*: Dickkopf WNT signalling pathway inhibitor 1 gene
DNA: Deoxyribonucleic acid
GAGs: Glycosaminoglycans
*GAPDH*: Glyceraldehyde-3-phosphate dehydrogenase protein
gDNA: Genomic DNA
GO: Gene ontology
GSEA: Gene set enrichment analysis
hBM-MSCs: Human bone marrow mesenchymal stromal cells
*HEY-1*: *Hairy/enhancer-of-split related with YRPW motif protein 1 gene*
H&E: Hematoxylin and eosin
ISCT: The international society for cellular therapy
*OCN*: Osteocalcin gene
*OPN*: Osteopontin gene
*ON*: Osteonectin gene
PBS: Phosphate-buffered saline
qPCR: Quantitative real-time polymerase chain reaction
rhBMP-2: Recombinant human bone morphogenetic protein 2
RNA: Ribonucleic acid
*RUNX2*: RUNX family transcription factor 2
*SOST*: Sclerostin gene
TCP: Tricalcium phosphate
WNT: Wingless-related integration site pathway
