# Supplementary figures and images for: rhBMP-2 induces terminal differentiation of human bone marrow mesenchymal stromal cells only by synergizing with other signals
Source: Stem Cell Res Ther. 2024 Apr 29;15:124. doi: 10.1186/s13287-024-03735-y (PMC11057131; doi:10.1186/s13287-024-03735-y)

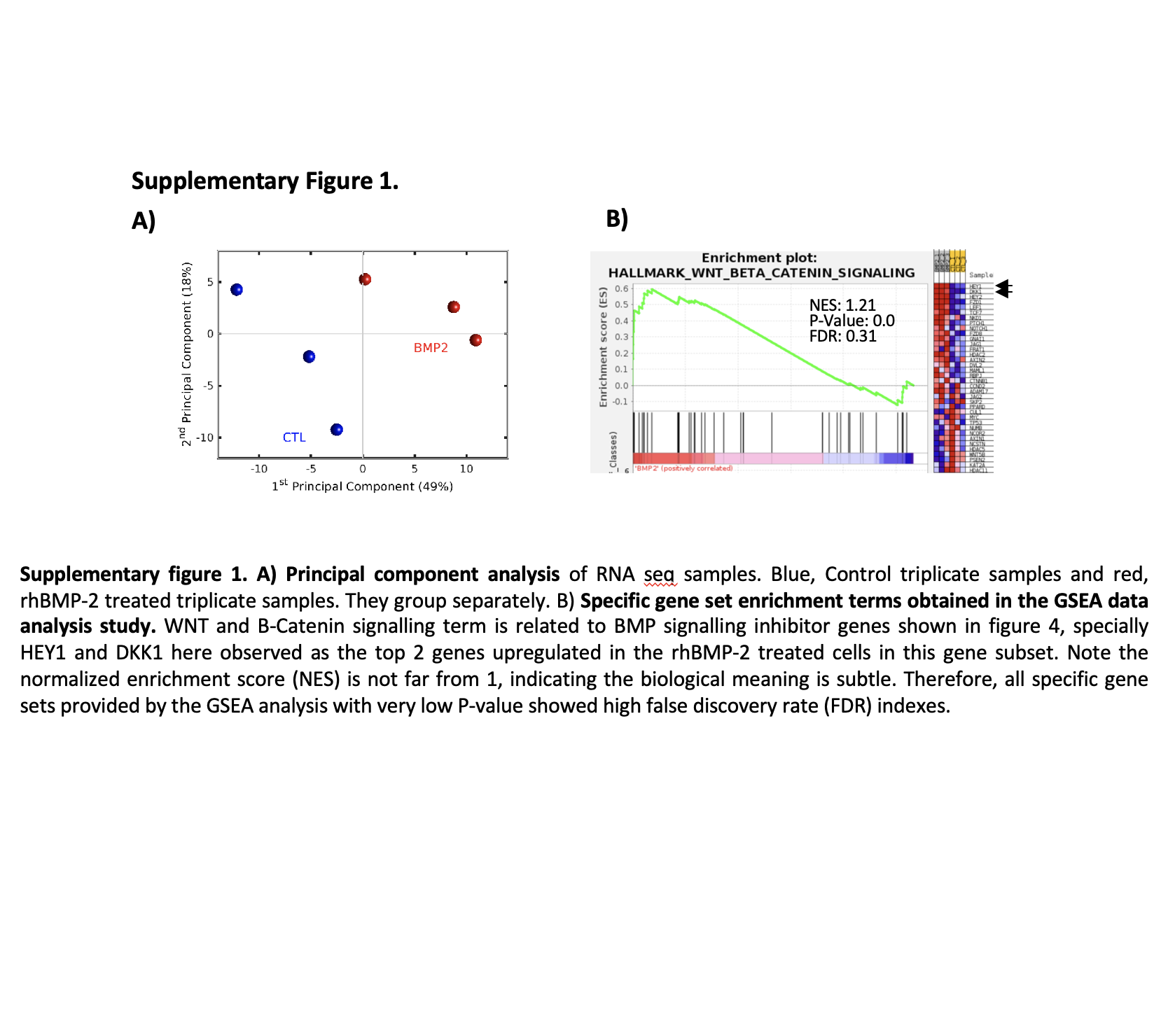

Supplement: Supplementary file 1 — Additional file 1: Figure 1. A Principal component analysis of RNA-seq samples. Blue, control triplicate samples; red, rhBMP-2-treated triplicate samples. The two groups were separated. All of them were from donor D24 hBM-MSCs. B Specific gene set enrichment terms obtained in the GSEA. The WNT and B-catenin signalling terms are related to the BMP signalling inhibitor genes shown in Fig. 4. Specifically, HEY1 and DKK1 were the top 2 genes upregulated in the rhBMP-2-treated cells in this gene subset. Note that the normalized enrichment score (NES) is not far from 1, indicating that the biological meaning is subtle. Therefore, all specific gene sets identified by GSEA with very low P values had high false discovery rate (FDR) indices. [file 13287_2024_3735_MOESM1_ESM.tiff]

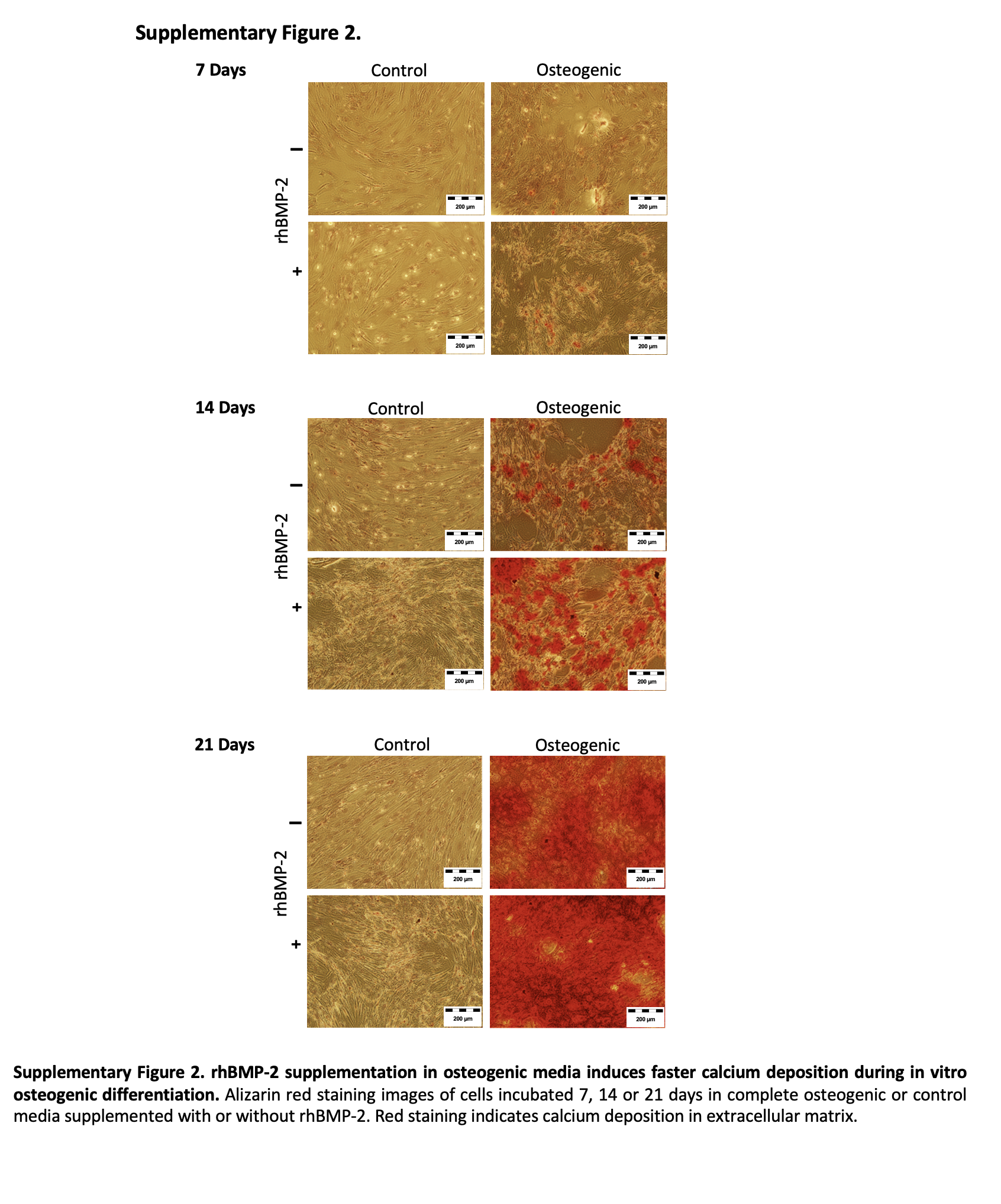

Supplement: Supplementary file 2 — Additional file 2: Figure 2. rhBMP-2 supplementation in osteogenic media induces faster calcium deposition during in vitro osteogenic differentiation. Alizarin red staining images of cells incubated for 7, 14 or 21 days in complete osteogenic or control media supplemented with or without rhBMP-2. Red staining indicates calcium deposition in the extracellular matrix. The data shown correspond to D19 hBM-MSCs. [file 13287_2024_3735_MOESM2_ESM.tiff]
