# Supplementary material for: rhBMP-2 induces terminal differentiation of human bone marrow mesenchymal stromal cells only by synergizing with other signals
Source: Stem Cell Res Ther. 2024 Apr 29;15:124. doi: 10.1186/s13287-024-03735-y (PMC11057131; doi:10.1186/s13287-024-03735-y)
Supplement: Supplementary file 3 — Additional file 3. Table of primers used in this study. [file 13287_2024_3735_MOESM3_ESM.docx]

**Supplementary table 1. Primers used in this study.**

| **Primer Name** | **Sequence** | **Tm** | **GC content** |
| --- | --- | --- | --- |
| *ALPL-F* | GGGAACGAGGTCACCTCCAT | 67.5 | 60 |
| *ALPL-R* | TGGTCACAATGCCCACAGAT | 66.6 | 50 |
| *RUNX2-F* | CGGCCCTCCCTGAACTCT | 66,8 | 66,6 |
| *RUNX2-R* | TGCCTGCCTGGGGTCTGTA | 69,2 | 63,1 |
| *OPN-F* | GTCTCAGGCCAGTTGCAGC | 66.3 | 63.1 |
| *OPN-R* | GGCACAGGTGATGCCTAGGA | 67.5 | 60 |
| *OCN-F* | GAAGCCCAGCGGTGCA | 67.9 | 68.7 |
| *OCN-R* | CACTACCTCGCTGCCCTCC | 67.2 | 68.4 |
| *ON-F* | GAAAGAAGATCCAGGCCCTC | 57.66 | 55 |
| *ON-R* | CTTCAGACTGCCCGGAGA | 58.3 | 61.11 |
| *LPL-F* | TTGTGAAATGCCATGACAAGTCT | 59.11 | 39.13 |
| *LPL-R* | CATGCCGTTCTTTGTTCTGTAGA | 59.25 | 43.48 |
| *LEP-F* | TCCCCTCTTGACCCATCTC | 57.68 | 57.89 |
| *LEP-R* | GGGAACCTTGTTCTGGTCAT | 57.41 | 50 |
| *PPARG-F* | CGGTTTCAGAAGTGCCTTG | 56.85 | 52.63 |
| *PPARG-R* | GGTTCAGCTGGTCGATATCAC | 58.53 | 52.38 |
| *FABP4-F* | TACTGAGATTTCCTTCATACTGGGC | 60,16 | 44 |
| *FABP4-R* | GCTCTCTCATAAACTCTCGTGGAAG | 60,96 | 48 |
| *SOX9-F* | GCGACGTCATCTCCAACATC | 65.8 | 55 |
| *SOX9-R* | TGGTCGGTGTAGTCGTACTG | 61.3 | 55 |
| *COL10A1-F* | ATGCTGCCACAAATACCCTTT | 64.3 | 42.8 |
| *COL10A1-R* | GGTAGTGGGCCTTTTATGCCT | 65 | 52.3 |
| *ACAN-F* | AAGTGCTATGCTGGCTGGTT | 57.2 | 50 |
| *ACAN-R* | GGTCTGGTTGGGGTAGAGGT | 58.5 | 60 |
| *COMP-F* | AACACGGTCACGGATGACGACTATG | 64,78 | 52 |
| *COMP-R* | CACAGAGCGTTCCGCAGCTGTTC | 66,59 | 66,59 |
